# Secreted Protein Acidic and Rich in Cysteine (SPARC) Induced by the Renin–Angiotensin System Causes Endothelial Inflammation in the Early Stages of Hypertensive Vascular Injury

**DOI:** 10.3390/ijms26094414

**Published:** 2025-05-06

**Authors:** Hiroe Toba, Mitsushi J. Ikemoto, Miyuki Kobara, Denan Jin, Shinji Takai, Tetsuo Nakata

**Affiliations:** 1 Laboratory of Clinical Pharmacology, Division of Pathological Sciences, Kyoto Pharmaceutical University, Kyoto 607-8412, Japan; kobara@mb.kyoto-phu.ac.jp (M.K.); tetsu@koto.kpu-m.ac.jp (T.N.); 2 Department of Pharmacology, Osaka Medical and Pharmaceutical University, Takatsuki-City 569-8686, Japan; denan.jin@ompu.ac.jp (D.J.); shinji.takai@ompu.ac.jp (S.T.); 3 Molecular Composite Physiology Research Group, Health and Medical Research Institute, National Institute of Advanced Industrial Science and Technology (AIST), Tsukuba 305-8566, Japan; ikemoto.mitsushi.gp@u.tsukuba.ac.jp; 4 Laboratory of Exercise Biochemistry and Neuroendocrinology, Institute of Health and Sport Sciences, University of Tsukuba, Tsukuba 305-8574, Japan

**Keywords:** extracellular matrix, secreted protein acidic and rich in cysteine, angiotensin II, hypertension, inflammation

## Abstract

Secreted protein acidic rich in cysteine (SPARC), one of the extracellular matrix proteins, is highly induced during inflammation. We investigated the pathophysiological regulation and role of SPARC in vascular inflammation in a rat model of hypertension created using deoxycorticosterone acetate (DOCA, 40 mg/kg/week, s.c.) and salt (1% in drinking water). DOCA–salt administration time-dependently increased systolic blood pressure during the 3-week treatment period, blunted endothelium-dependent vasodilation, and increased monocyte chemoattractant protein-1 (MCP-1) and lectin-like oxidized low-density lipoprotein receptor-1 (LOX-1) expression in the aorta. SPARC expression transiently increased until week 2 in the DOCA–salt rat aorta. Interestingly, aortic SPARC levels correlated with blood pressure and the levels of MCP-1 and LOX-1 during 0–2 weeks. The AT_1_ receptor blocker, losartan, suppressed the overexpression of SPARC, and in vitro treatment with angiotensin II enhanced the production of SPARC in rat aortic endothelial cells. Exposure to recombinant SPARC protein induced overexpression of MCP-1 and LOX-1 mRNA in endothelial cells. Bioactive forms of a disintegrin and metalloproteinase with thrombospondin type 1 motif (ADAMTS1), excessive activation of which contributes to pathological states and overexpression of which is reported to be induced by SPARC, were increased in the DOCA–salt rat aorta. These results suggest that SPARC is induced by the vascular renin–angiotensin system and causes inflammation in the early stages of hypertensive vascular injury, and that activation of ADAMTS1 might be related to the proinflammatory effects of SPARC.

## 1. Introduction

Hypertension is one of the major risk factors for vascular diseases. Endothelial dysfunction is the initial stage of hypertensive vascular injury and leads to a chronic inflammatory process and, eventually, plaque formation and rupture when complicated with other risk factors, such as diabetes and dyslipidemia. These phenomena drastically increase the risk of onset and progression of vascular damage [1,2,3]. Atherosclerosis is a leading cause of death worldwide, particularly in industrial countries [4]. Unfortunately, there are no therapeutic strategies to directly suppress endothelial damage and plaque formation.

For a long time, the extracellular matrix (ECM) has been considered as mainly serving as the scaffold between cells. However, it also contributes to the regulation of various cellular functions beyond its constitutional properties. Secreted protein acidic and rich in cysteine (SPARC), one of the ECMs, plays roles in development and tissue formation, and, in physiological settings, its expression is only localized in remodeling tissue, such as bones and intestinal epithelium [5,6]. Expression of SPARC is upregulated in damaged tissues in proportion to the degree of inflammation [7]. On the other hand, SPARC gene deletion has been shown to attenuate inflammatory symptoms in animal models of dextran sodium sulfate-induced colitis, hypertensive renal injury, and lipopolysaccharide-induced footpad edema [8,9,10]. In patients with gestational diabetes mellitus, plasma SPARC levels positively correlate with white blood cell numbers and high-sensitive C-reactive protein, both of which are markers of inflammation [11]. These observations indicate that SPARC is a proinflammatory factor.

Recently, we reported that the AT_1_ receptor blocker, losartan, suppresses overexpression of SPARC in the kidneys in a rat model of deoxycorticosterone acetate (DOCA) and high salt-induced hypertension, and that angiotensin II treatment triggers SPARC induction in rat renal fibroblast NRK-49F cells [12]. Activation of the local tissue renin–angiotensin system is well-known as playing pivotal roles in exacerbating atherosclerosis via many mechanisms, including endothelial dysfunction and proinflammatory gene expression, which ultimately leads to cardiovascular and renal events [13,14]. Hence, investigation of the correlation between SPARC and the vascular renin–angiotensin system will likely provide beneficial information for the treatment of hypertensive vascular diseases. Our recent studies indicate that a disintegrin and metalloproteinase with thrombospondin type 1 motif (ADAMTS1) likely mediates the pathological roles of SPARC in aging hearts, hypertensive renal injury, and renal ischemia/reperfusion injury [12,15,16]. The aim of the present study was to examine the hypothesis that SPARC might exert proinflammatory effects in DOCA–salt hypertensive rats, and to evaluate the contribution of the vascular renin–angiotensin system and ADAMTS1 to this process. This is the first description of the novel aspects of SPARC as an early effector of vascular inflammation in the setting of hypertension.

## 2. Results

### 2.1. Physiological and Biochemical Data of Rats Treated with DOCA–Salt

Physiological data of the rats are summarized in Table 1. Body weight was higher in the 1w, 2w, and 3w groups than in the 0w group. Systolic blood pressure showed significant time-dependent increases from week 1. Decreases in heart rates were observed in weeks 1 and 2. Urinary nitric oxide metabolite (NOx; NO_2_^−^+NO_3_^−^) concentrations started to decrease from week 2, and were significantly decreased at week 3, suggesting a time-dependent decline in vascular endothelial cell function. The concentrations of malondialdehyde, a major lipid oxidation product and an index of oxidative stress, tended to increase in week 3.

### 2.2. Endothelial Cell Dysfunction and Vascular Inflammation Occurred in the DOCA–Salt Rat Aorta

**Figure 1:**
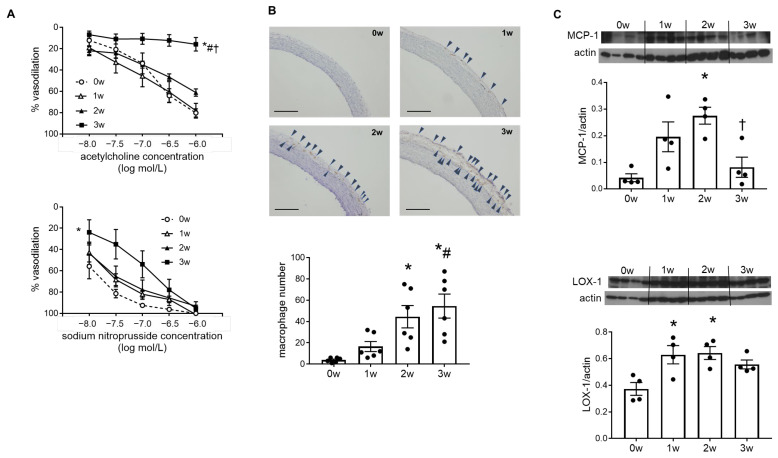
Endothelium-dependent dilatory responses were blunted, and inflammation was increased in deoxycorticosterone acetate (DOCA)–salt rat aortas. (**A**) Concentration–response curves for acetylcholine (**upper**) and sodium nitroprusside (**lower**) of the aortic rings from rats treated with DOCA and salt for 0 weeks (0w), 1 week (1w), 2 weeks (2w), or 3 weeks (3w). (**B**) Immunohistochemical staining for macrophages of representative sections of the aorta from rats treated with DOCA and salt for 0w, 1w, 2w, or 3w. The arrowheads indicate Ki-M2R-positive cells. Bars indicate 200 μm (**upper**). Number of Ki-M2R-positive cells that infiltrated into the aortic vessel (**lower**). (**C**) Monocyte chemoattractant protein-1 (MCP-1, **upper**) and lectin-like oxidized low-density lipoprotein receptor-1 (LOX-1, **lower**) protein expression in the aortas of rats in the 0w, 1w, 2w, and 3w groups. Bands were quantified by densitometry. Values are means ± SEM (*n* = 6/group for (**A**,**B**), *n* = 4/group for (**C**)). * *p* < 0.05 vs. 0w, # *p* < 0.05 vs. 1w, † *p* < 0.05 vs. 2w.

Dose-dependent dilatory responses of the aorta to acetylcholine were markedly blunted in the 3w group, compared to the 0w, 1w, and 2w groups. Arterial responses to sodium nitroprusside seemed to be weaker in the 3w group than the other groups at the lower doses, but there were no significant differences in dilatory responses at the maximum sodium nitroprusside dosage (Figure 1A). Together with the results of urinary NOx levels, these results indicate that vascular endothelial cell function was impaired in DOCA–salt hypertensive rats at week 3.

Endothelial injury leads to inflammatory cell recruitment into the vascular wall, and inflammation plays a central role in the pathogenesis of atherosclerosis [2,3]. In this study, the number of macrophages in the aortic adventitia tended to increase from week 1 and showed significant increases from week 2, as seen by immunohistochemical analysis (Figure 1B). Monocyte chemoattractant protein-1 (MCP-1) is a potent chemokine for monocytes, and induction of MCP-1 occurs at the sites of lesion formation in the endothelial cells in the early stages of atherosclerosis [17]. We observed significantly higher MCP-1 expression levels in 2-week DOCA–salt rats than in 0-week normotensive rats, with time-dependent increases until week 2. Lectin-like oxidized low-density lipoprotein receptor-1 (LOX-1), which was originally identified as a scavenger receptor for oxidized LDL in vascular endothelial cells, binds not only to oxidized LDL, but also to other ligands, such as activated platelets and apoptotic cells [18]. Expression of LOX-1 is upregulated in proatherogenic conditions, including hypertension, and LOX-1 plays a deteriorative role in various stages of atherosclerosis [19]. In this study, LOX-1 expression in the aorta was higher in 1- and 2-week DOCA–salt rats than in 0-week rats (Figure 1C).

### 2.3. Overexpression of SPARC and ADAMTS1 Was Induced in the Early Stages of Vascular Injury in DOCA–Salt Rats

**Figure 2:**
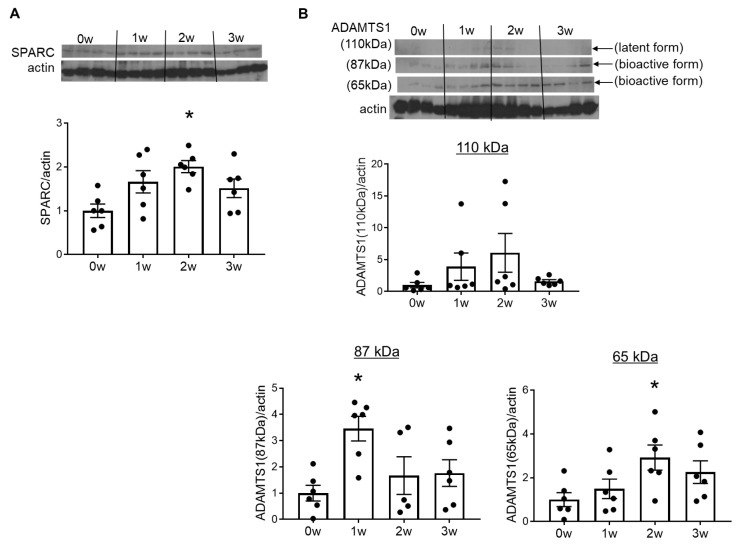
Secreted protein acidic and rich in cysteine (SPARC) and a disintegrin and metalloproteinase with thrombospondin type 1 motif (ADAMTS1) protein expression in deoxycorticosterone acetate (DOCA)-salt rat aortas increased in the early stages of vascular injury. The protein expression of SPARC (**A**) and ADAMTS1 (**B**) in the aortas of rats treated with DOCA and salt for 0 weeks (0w), 1 week (1w), 2 weeks (2w), or 3 weeks (3w). Bands were quantified by densitometry. Values are means ± SEM (*n* = 5–6/group). * *p* < 0.05 vs. 0w.

Western blotting showed an increase in SPARC protein expression from week 1 and significant increases at week 2. The upregulation of SPARC peaked at week 2, with no significant differences between the 0w and 3w groups (Figure 2A). These results suggest that SPARC expression was upregulated in the early stages of vascular inflammation and prior to endothelial dysfunction.

ADAMTS1 maintains ECM turnover by cleaving particular ECM components, such as collagen I, aggrecan, and versican. The latent form of ADAMTS1 (110 kDa) is processed to the 87-kDa bioactive form, which is further cleaved by matrix metalloproteinases to obtain another bioactive form (65 kDa) containing the thrombospondin motif [20]. Our recent studies provided evidence to demonstrate that SPARC promotes cardiac and renal collagen deposition via the production of ADAMTS1 [12]. Therefore, we also investigated changes in ADAMTS1 expression in the DOCA–salt treated rat aorta in the present study. No significant differences were observed in the 110-kDa form of ADAMTS1 among all groups, while there were slight and time-dependent increases until week 2. One- and 2-week DOCA–salt rats showed higher expressions of the 87-kDa and 65-kDa forms, respectively, than 0-week normotensive rats, indicating that ADAMTS1 is activated in the aorta of DOCA–salt hypertensive rats (Figure 2B).

**Figure 3:**
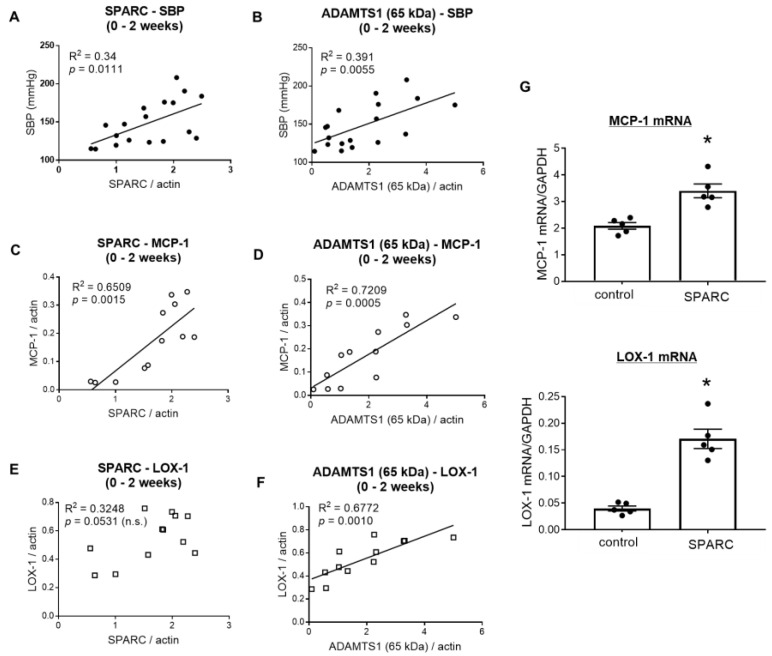
Expression of secreted protein acidic and rich in cysteine (SPARC) and a disintegrin and metalloproteinase with thrombospondin type 1 motif (ADAMTS1, the 65-kDa bioactive form) correlated with systolic blood pressure and vascular inflammation in deoxycorticosterone acetate (DOCA)-salt hypertensive rats in the first two weeks of DOCA–salt treatment, and SPARC treatment induced vascular endothelial cell inflammation. Correlations between SPARC (**A**,**C**,**E**) or ADAMTS1 (**B**,**D**,**F**) expression and systolic blood pressure (**A**,**B**), monocyte chemoattractant protein-1 (MCP-1, **C**,**D**), and lectin-like oxidized low-density lipoprotein receptor-1 (LOX-1, **E**,**F**) expression during 0–2 weeks of DOCA–salt treatment. (**G**) The mRNA expression of MCP-1 (**upper**) and LOX-1 (**lower**) in cultured rat aortic endothelial cells stimulated with the control protein or the SPARC protein (100 nmol/L) for 24 h. Values are means ± SEM (*n* = 5/group for (**G**)). * *p* < 0.05 vs. control (**G**).

To explore how the changes in SPARC and ADAMTS1 correlated with physiological and biological parameters, we investigated the association between the expression of these molecules and systolic blood pressure, MCP-1 expression, and LOX-1 expression (Figure 3A–F). Since the expression of SPARC and ADAMTS1 peaked at 2 weeks, we compared the changes of these molecules with systolic blood pressure and the expression of MCP-1 and LOX-1 during 0–2 weeks after DOCA–salt treatment. SPARC and ADAMTS1 (the 65-kDa bioactive form) expression showed a positive correlation with time-dependent increases in systolic blood pressure and MCP-1 expression until 2 weeks after DOCA–salt treatment (Figure 3A–D). These observations suggest that the SPARC and ADAMTS1 expression induced in the DOCA–salt rats played some role in blood pressure elevation and vascular inflammation until 2 weeks. On the other hand, SPARC levels had no correlation with vascular LOX-1 expression (Figure 3E), while the 65-kDa form of ADAMTS1 and LOX-1 levels significantly correlated with each other (Figure 3F). While MCP-1 promotes vascular injury in the early stages of atherosclerosis, LOX-1 reportedly mediates not only endothelial dysfunction and inflammatory cell infiltration, but also atherosclerotic plaque formation, thrombogenesis, and platelet aggregation [21,22]. This suggests that, compared to ADMATS1, SPARC might contribute more to the earlier stages of vascular inflammation.

### 2.4. SPARC Induced Proinflammatory Gene Expression in Vascular Endothelial Cells

To investigate the role of SPARC, which was transiently increased in the early stages of hypertensive vascular injury, on vascular inflammation, we treated cultured endothelial cells with recombinant SPARC protein and measured the expression of MCP-1 and LOX-1 mRNA. SPARC treatment significantly enhanced the levels of MCP-1 and LOX-1 mRNA, suggesting that SPARC exhibits proinflammatory effects (Figure 3G), although experiments with negative controls, such as heat-inactivated recombinant SPARC protein, are needed to corroborate the current in vitro findings.

### 2.5. Activation of the Vascular Renin–Angiotensin System Increased SPARC Expression in DOCA–Salt Rats

**Figure 4:**
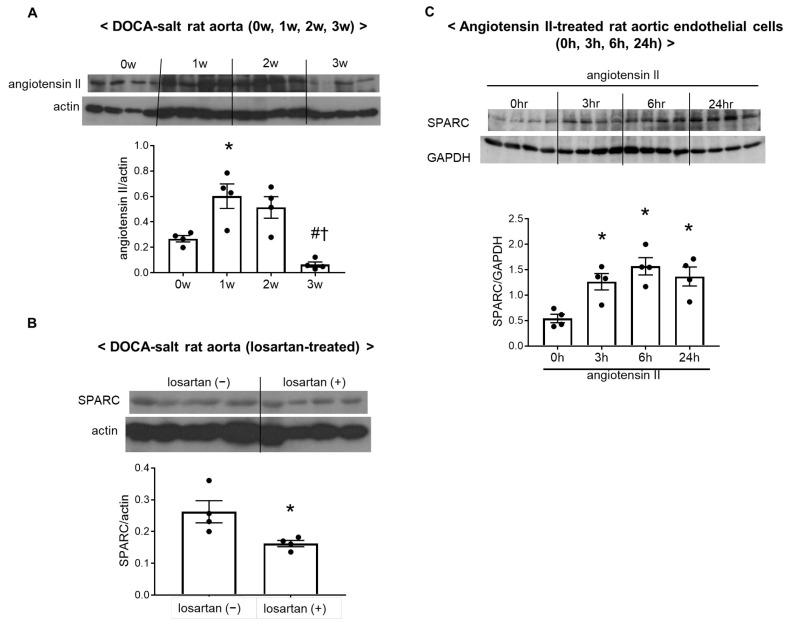
The vascular renin–angiotensin system triggered secreted protein acidic rich in cysteine (SPARC) overexpression. (**A**) The protein levels of angiotensin II in the aorta of rats treated with deoxycorticosterone acetate (DOCA) and salt for 0 weeks (0w), 1 week (1w), 2 weeks (2w), or 3 weeks (3w). (**B**) The protein expression of SPARC in the aortas of losartan-nontreated or -treated 2-week DOCA–salt rats. (**C**) The protein expression of SPARC in cultured rat aortic endothelial cells treated with angiotensin II (100 nmol/L) for 0 h, 3 h, 6 h, or 24 h. Immunoblot bands were quantified by densitometry. Values are means ± SEM (*n* = 4/group for (**A**–**C**)). * *p* < 0.05 vs. 0w, # *p* < 0.05 vs. 1w, † *p* < 0.05 vs. 2w (**A**). * *p* < 0.05 vs. the losartan-nontreated group (**B**), or 0 h (**C**).

Angiotensin II is the principal effector of the renin–angiotensin system. In this study, aortic expression of angiotensin II was significantly higher in 1-week DOCA–salt rats than in 0-week normotensive rats. This increase in angiotensin II levels in DOCA–salt treated rats was suppressed in the 3-week group (Figure 4A).

To investigate whether the vascular renin–angiotensin system is placed upstream of SPARC induction, DOCA–salt rats were treated with the AT_1_ receptor blocker, losartan, for 2 weeks, because SPARC levels peaked during this treatment period. Elevation of SPARC expression levels in DOCA–salt rats was blunted in the losartan-treated group (Figure 4B). The present results indicated that SPARC was induced by activation of the vascular renin–angiotensin system.

To confirm our hypothesis, angiotensin II was added to cultured rat aortic endothelial cells. In vitro treatment with angiotensin II for 3, 6, and 24 h caused the overexpression of SPARC in cultured rat aortic endothelial cells, supporting the hypothesis that activation of the vascular renin–angiotensin system induces SPARC expression in the aorta (Figure 4C).

## 3. Discussion

The present study demonstrated that (1) aortic SPARC expression transiently increased in DOCA–salt hypertensive rats in the early stages of vascular inflammation, when the endothelium-dependent vasodilatory response was still preserved; (2) the levels of SPARC expression corelated with systolic blood pressure and aortic MCP-1 expression, (3) activation of the vascular renin–angiotensin system triggered the overexpression of SPARC; and (4) treatment with SPARC induced increases in MCP-1 and LOX-1 mRNA in cultured aortic endothelial cells. This study provides the first evidence that activation of the vascular renin–angiotensin system triggers SPARC overexpression, and that SPARC facilitates vascular inflammation.

This study showed that SPARC expression was induced in the early stages of vascular inflammation (until 2 weeks) in DOCA–salt hypertensive rats. Inducible expression of SPARC in DOCA–salt rats indicates its pathogenic role in the initiation and progression of chronic inflammation in the vascular tissue. Similar to our observations in the present study, SPARC content reportedly starts to increase from week 2 in the DOCA–salt rat kidney, along with a significant increase in the number of infiltrating macrophages [12]. Analysis of the correlation between SPARC expression and blood pressure and vascular inflammation suggested that the increment in SPARC might induce hypertension and vascular injury. A recent study reported that serum SPARC levels are higher in hypertensive individuals compared to non-hypertensive controls, and that blood pressure values increased proportionately with increases in serum SPARC levels [23]. The same report demonstrated that in vivo injection of SPARC increased systolic blood pressure in mice, suggesting that SPARC induces hypertension. In addition, ex vivo treatment with SPARC significantly and dose-dependently impaired the acetylcholine-induced dilatory reaction in the murine thoracic aorta [23]. Therefore, the observed overexpression of SPARC in this study might have mediated the pathogenesis of hypertension and vascular injury, at least in part, in the early stages of the diseases. These observations also suggest that SPARC-induced vascular endothelial cell dysfunction might be one of the mechanisms for hypertension. In addition, the remodeling properties of SPARC might play a role in the correlation between SPARC and blood pressure elevation. In previous studies in human subjects and mice with pulmonary hypertension, SPARC was shown to be overexpressed in lungs, and SPARC suppression using adenovirus vector attenuated cardiac and hemodynamic disorders [24]. This report also demonstrates that SPARC, released from pulmonary microvascular endothelial cells, triggers proliferation of pulmonary arterial smooth muscle cells [24]. However, precise causality between SPARC and blood pressure needs further validation. Recombinant SPARC protein treatment increases the expression of proinflammatory M1 macrophage markers, such as chemokine (C-C motif) ligand 5 (Ccl5), Ccl3, tumor necrosis factor-α, and interleukin-12, and decreases anti-inflammatory M2 markers, such as arginase 1 and mannose receptor C type 1 [7]. Macrophage M1 > M2 polarization might be one of the mechanisms by which SPARC functions as a driver of inflammation. SPARC binds to certain receptors, including vascular cell adhesion molecule-1 (VCAM-1) and β1-integrin [25,26]. Damaged endothelial cells express VCAM-1, which facilitates monocyte infiltration into the arterial wall. Although further studies are needed to clarify this, the binding of SPARC to VCAM-1 might mediate the induction of proinflammatory molecules. A recent study demonstrated that SPARC also aggravates blood–brain-barrier disruption after subarachnoid hemorrhage via αvβ3-integrin signaling pathway-mediated endothelial cell injury [27]. Hence, future studies into the mechanisms of SPARC-induced vascular endothelial cell injury will help to provide a novel preventive strategy against hypertensive vascular injury.

The AT_1_ receptor blocker, losartan, inhibited overexpression of SPARC in DOCA–salt hypertensive rats. DOCA–salt-treated animals are widely used in experiments on hypertension and hypertension-related diseases. Chronic treatment with DOCA and salt results in volume retention, which causes malignant hypertension with a low plasma renin level [28,29]. In this model, despite inactivation of the systemic renin–angiotensin system, the tissue renin–angiotensin system is activated in vascular tissue, serving as a major source of vascular injury [30,31]. Thus, DOCA–salt hypertensive animals are useful for investigating the roles of the local (tissue) renin–angiotensin system, because the secondary effects of blood pressure-lowering actions caused by AT_1_ receptor blockers or angiotensin-converting enzyme inhibitors are excluded. In the present study, 2-week treatment with losartan had no effects on systolic blood pressure (losartan (−) group 180 ± 4.8 mmHg vs. losartan (+) group 171 ± 10.8 mmHg; n.s.), although losartan treatment decreased vascular SPARC expression (Figure 4B). The observed results in losartan-treated rats and angiotensin II-treated aortic endothelial cells suggest that the vascular renin–angiotensin system is upstream of the induction of SPARC. A previous study reported that both the angiotensin-converting enzyme inhibitor, ramipril, and the AT_1_ receptor blocker, valsartan, reduced SPARC overexpression in subtotal nephrectomized rats, suggesting that angiotensin II-induced SPARC increments are mediated by the AT_1_ receptor [32]. Therefore, we used the AT_1_ receptor blocker losartan in the present study. We measured SPARC expression, but not other factors related to vascular inflammation, in the losartan-treated DOCA–salt group. The vascular renin–angiotensin system plays pivotal roles in vascular inflammation in the setting of common diseases, such as hypertension, diabetes and dyslipidemia, and our previous study demonstrated that treatment with losartan attenuated vascular inflammation and endothelial dysfunction in hypertensive and normotensive subtotal nephrectomized rats [33,34]. Therefore, we presumed that vascular alteration would be improved by losartan in DOCA–salt hypertensive rats. To date, no clinical studies have corroborated the proposed association between SPARC and the renin–angiotensin system. The present study might provide future options for selecting SPARC inhibitors in patients with hypertension and hypertensive organ damage who are not eligible to receive renin–angiotensin system inhibitors because of side effects or contraindications. A recent experimental study using human umbilical vein endothelial cells reported that SPARC causes inflammation by reducing angiotensin-converting enzyme 2 (ACE2) [23]. ACE2 degrades angiotensin II, producing angiotensin (1–7), which is known to counteract the effects of the AT_1_ receptor-mediated angiotensin II. Corroboration of the relationship between SPARC and the renin–angiotensin system in clinical studies is anticipated. In healthy individuals, plasma SPARC levels range from 0.1–0.8 μg/mL (≈2.5–20 nmol/L), increasing to 1.5–10 μg/mL (≈37.5–250 nmol/L) in neoplastic and inflammatory conditions [35,36,37]. We observed that treatment with SPARC protein (100 nmol/L) caused the upregulation of MCP-1 and LOX-1. Many previous studies using SPARC knockout animals have indicated the pro-inflammatory roles of SPARC [8,9,10]. Furthermore, a recent study reported that SPARC treatment, either in vivo (1 μg/kg, intraperitoneally) or ex vivo (500–1000 ng/mL), significantly blunted acetylcholine-induced endothelium-dependent vasodilatory responses in the thoracic aorta isolated from mice [23]. Therefore, we presume that SPARC might be a proinflammatory factor in hypertensive vascular injury.

ADAMTS1 is widely and constitutively distributed in various tissues, and its excessive activation is reported to contribute to pathological states [38]. In the present study, bioactive forms of ADAMTS1 were enhanced in the DOCA–salt rat aorta, suggesting that ADAMTS1 might play a role in vascular injury. Indeed, transgenic overexpression of ADAMTS1 caused inflammatory cell infiltration into extracellular spaces and severe remodeling after carotid artery ligation in ApoE-deficient atherosclerosis model mice [39]. In addition, we previously reported that SPARC induces ADAMTS1 protein production, and that cotreatment with anti-ADAMTS1 neutralizing antibody and SPARC suppresses SPARC-induced collagen I overexpression [15]. These findings support the hypothesis that SPARC-mediated vascular injury might be caused, at least in part, by the activation and/or production of ADAMTS1. However, further investigation into the association between SPARC and ADAMTS1 is needed to clarify the pathway by which SPARC induces ADAMTS1 expression. Reportedly, stimulation of αvβ3-integrin by SPARC causes activation of matrix metalloproteinase-9 (MMP-9) via the mitogen-activated protein kinase (MAPK) pathway, leading to endothelial cell damage [27]. Activation of the αvβ3-integrin/MAPK pathway might mediate not only MMP-9 but also ADAMTS1 activation. Further studies using inhibitors of integrin αVβ3 and MAPK should be performed to confirm this hypothesis.

There are some limitations to this study. First, to confirm the relevance of SPARC in hypertension and hypertension-related vascular injury, additional validation in alternative hypertensive animal models, such as spontaneously hypertensive rats (SHR), N-nitro-L-arginine methyl ester-induced hypertensive animals, and Dahl-salt sensitive hypertensive rats, is needed. In particular, since SHR is a well-known animal model of human essential hypertension [40], future investigation into SHR is essential. Second, the current study did not clarify the precise mechanisms by which SPARC and ADAMTS1 contribute to vascular inflammation and endothelial dysfunction. As described above, macrophage polarization into proinflammatory M1 phenotype, VCAM-1- and integrins-mediated endothelial cell damage, and inflammatory cell infiltration following ADAMTS1-induced excessive ECM degradation might play roles in SPARC-induced vascular injury [7,25,26,39]. In addition, ADAMTS1 reportedly exerts pathophysiological roles via its thrombospondin-motifs independent of its enzymatic properties [41]. Clinical studies showing the correlation between plasma SPARC levels and blood pressure and inflammatory markers in plasma indicate that elevation of SPARC and ADAMTS1 might be a possible predictive diagnostic marker for vascular injury in the setting of hypertension [11,23]. SPARC might increase first, followed by subsequent upregulation/activation of ADAMTS1, suggesting that they might serve as stage-dependent biomarkers. Furthermore, our recent study demonstrated that tail vein injection of small interfering RNA targeting SPARC attenuated ischemia/reperfusion-induced renal dysfunction, inflammation, fibrosis and ADAMTS1 overexpression, indicating a novel therapeutic strategy of SPARC suppression in renal disease and a role of ADAMTS1 as a mechanism of SPARC-induced renal injury [16]. Third, we did not evaluate the regulatory mechanisms of dynamic changes in SPARC expression. Possible mechanisms might include regulatory or compensatory feedback that occurs in the later stages of vascular damage. Some growth factors, including transforming growth factor-β, are reportedly involved in the regulation of SPARC expression [42]. Changes in the balance of growth factor components in acute and chronic stages of vascular injury might affect the temporal peak in the early stage of vascular injury and subsequent decline in SPARC expression. Transient increases in SPARC, which correlate with collagen deposition in the initial stage of interstitial fibrosis, have been reported in an animal model of nephritis [43]. Our current findings, taken together with the previous report, suggest that SPARC induction might be associated with the onset of vascular injury rather than its progression. Further investigation into the time-dependent changes of SPARC over longer periods and more chronic stages of vascular injury is needed.

## 4. Materials and Methods

### 4.1. Animals

All animal procedures were performed at Kyoto Pharmaceutical University based on the National Research Council’s and National Institute of Health guidelines, and were approved by the Institutional Animal Care and Use Committee of Kyoto Pharmaceutical University (CPCO-19-001, A23-015-01). Male Wistar/ST rats (Shimizu Laboratory Supply, Kyoto, Japan) were housed in an environmentally controlled room with a 12-h light/dark cycle, given standard rodent chow and tap water ad libitum, and acclimated to handling. Rats weighing 150 g and 230 g were used for the following DOCA–Salt Treatment and Cell Culture experiments, respectively.

### 4.2. DOCA–Salt Treatment

Right unilateral nephrectomy was performed under anesthesia (0.375 mg/kg medetomidine, 2.0 mg/kg midazolam, and 2.5 mg/kg butorphanol tartrate, i.p.), and rats were randomly divided into five groups; 0w, 1w, 2w, 3w, and 2w-losartan groups (*n* = 82, in total). After a one-week recovery period, DOCA (40 mg/kg/week, s.c.) and salt water (1% NaCl in drinking water) were administered for 0, 1, 2, or 3 weeks, respectively, in the 0w, 1w, 2w and 3w groups. Rats in the 2w-losartan group received the AT_1_ receptor blocker, losartan (MSD K.K., Tokyo, Japan), at the dose of 30 mg/kg/day by gavage for 2 weeks from the beginning of the DOCA–salt treatment. Systolic blood pressure and heart rates were measured by the tail-cuff method (BP-98A-L, Softron, Tokyo, Japan), and urine samples were collected using metabolic cages. Rats were euthanized under anesthesia (0.375 mg/kg medetomidine, 2.0 mg/kg midazolam, and 2.5 mg/kg butorphanol tartrate, i.p.) at the end of their study periods, based on their respective group allotments, and their blood samples and thoracic aortas were collected.

### 4.3. Urinary NOx and Plasma Malondialdehyde Levels

Urinary levels of NOx, the stable end products of nitric oxide (NO), were measured using a high-performance liquid chromatography–Griess system (ENO-15, Eicom, Japan), as described previously [44]. Plasma malondialdehyde concentrations were measured using a commercially available kit (Cayman Chemical, Ann Arbor, MI, USA).

### 4.4. Vasodilatory Responses

Vasodilatory responses were measured using an isometric transducer, as described previously [44]. Briefly, isolated aortic tissues were dissected from the adherent fat and connective tissue in 37 °C Krebs–Ringer solution, taking extreme care to avoid stretching of the tissue, and 3 mm-aortic rings were mounted on two parallel and horizontal wires inserted into the lumen of the rings in Krebs–Ringer solution, which was bubbled with a 95% O_2_–5% CO_2_ and maintained at 37 °C. One hook was connected to strain gages which were coupled to a polygraph for the recording of isometric tension. To evaluate endothelium-dependent and -independent dilation, acetylcholine or a nitric oxide donor, sodium nitroprusside (10^−8^ to 10^−6^ mol/L), was added cumulatively to the aortic rings after pre-contraction with a submaximal concentration of phenylephrine (10^−6^ mol/L).

### 4.5. Immunohistochemistry

Dissected aortic tissues were embedded in a freezing compound and snap-frozen. Cryosections (5 µm) were fixed in 4% phosphate-buffered formaldehyde and incubated in 0.3% hydrogen peroxide to block endogenous peroxide activity. Anti-rat pan-macrophages antibody (1:100, Ki-M2R, BMA Biomedicals, Augst, Switzerland) was used to detect macrophages by the avidin-biotin complex method using 3,3′-diaminobenzidine (Sigma-Aldrich, St. Louis, MO, USA) with hematoxylin counterstaining. The number of positively stained cells per total adventitial area was determined.

### 4.6. Western Blotting

Frozen aortas were homogenized in ice-cold Tris buffer (5 mmol/L, pH 7.4) containing protease inhibitors. The homogenates were centrifuged for 10 min at 100× *g*, and supernatants were collected. Proteins were mixed with sample buffer containing β-mercaptoethanol, boiled for 5 min, separated with sodium dodecyl sulfate-polyacrylamide gel electrophoresis, and then electro-transferred to PVDF membranes. The membranes were incubated overnight with primary antibodies for MCP-1 (1:500, Abcam, Cambridge, UK), LOX-1 (1:1000, Abcam), SPARC (1:300, Abnova, Taipei, Taiwan), ADAMTS1 (1:500, Bioss antibodies, Woburn, MA, USA), angiotensin II (1:1000, Absolute Antibody, Wilton, UK), and actin (1:5000, Merck Millipore, Darmstadt, Germany), and were then incubated with appropriate horseradish peroxidase-conjugated secondary antibodies (Cell Signaling Technology, Danvers, MA, USA; Promega, Madison, WI, USA). Bands were detected by enhanced chemiluminescence (Cytiva, Tokyo, Japan).

### 4.7. Cell Culture

Rat aortic endothelial cells were isolated from rats (*n* = 15 in total) by a primary explant technique, and cultured in Dulbecco’s modified Eagle’s medium (DMEM, Nacalai Tesque Inc., Kyoto, Japan) supplemented with endothelial cell growth supplement (100 μg/mL, Corning, Glendale, AZ, USA) using collagen I-coated culture dishes (35 mm, Corning) [37]. The cells were transferred to culture dishes without collagen I-coating and incubated in DMEM supplemented with 15% fetal bovine serum (Gibco, Thermo Fisher Scientific, Waltham, MA, USA), antibiotics (Gibco), and antimycotics (Gibco) at 37 °C under humidified 5% carbon dioxide conditions. The cells were maintained by passaging using 35-mm cell culture dishes when confluence was >80%, and cells from passage 3 were used for the experiments.

The cells were treated with recombinant SPARC protein (100 nmol/L) or control protein (100 nmol/L) for 24 h. The recombinant proteins were synthesized and purified at the National Institute of Advanced Industrial Science and Technology (AIST), as previously described [38]. Cells were used for RNA extraction as described below.

In a separate experiment, the cells were treated with angiotensin II (100 nmol/L, Sigma-Aldrich) for 0, 3, 6, or 24 h. Angiotensin II-treated cells were lysed in ice-cold lysis buffer, and Western blotting was performed to investigate the protein expression of SPARC. The amount of each SPARC band was normalized to the densitometry of the housekeeping protein, glyceraldehyde-3-phosphate dehydrogenase (GAPDH; 1:1000, Abcam).

### 4.8. RNA Extraction and Real-Time Quantitative Reverse Transcription-Polymerase Chain Reaction

Total RNA was isolated from cultured endothelial cells using the ISOGEN II reagent (Nippon Gene, Tokyo, Japan), and complementary DNA was synthesized using the PrimeScript^TM^ RT reagent kit (Takara Bio Inc., Kusatsu, Japan). To assess the mRNA expression of MCP-1 and LOX-1, quantitative real-time polymerase chain reaction tests were performed using TB Green Premix Ex TaqII (Takara) and the Thermal Cycler Dice Real Time System (Takara). The polymerase chain reaction primer sequences used are listed in Table 2. The relative mRNA expression of each target molecule was calculated by normalization of the threshold cycle (Ct) values of the target genes to the Ct values of the housekeeping gene GAPDH (Takara).

### 4.9. Statistical Analysis

Data were expressed as the means ± SEM. A one-way ANOVA followed by Tukey’s multiple comparisons test was used for comparison among the 0w, 1w, 2w, and 3w groups in the macrophage number and the expression of MCP-1, LOX-1, SPARC, ADAMTS1, and angiotensin II and among the 0 h, 3 h, 6 h, and 24 h groups in the expression of SPARC. Two group comparisons between the control and SPARC groups and between the losartan (+) and losartan (−) groups were performed using an unpaired *t*-test. The acetylcholine or sodium nitroprusside concentration-response curves of the different groups (vasodilatory responses in the 0w, 1w, 2w, and 3w groups) were compared by two-way repeated measures ANOVA followed by Tukey’s multiple comparisons test. Pearson’s correlation coefficient was assessed to calculate correlations between variables (between SPARC and SBP, MCP-1, or LOX-1; between ADAMTS1 (65kDa) and SBP, MCP-1, or LOX-1). *p* < 0.05 was considered as being statistically significant. Statistical analyses were performed using the GraphPad Prism 7 software.

## 5. Conclusions

Based on our results, we concluded that SPARC correlates with blood pressure and augments vascular inflammation in the setting of hypertension. In this process, activation of the vascular renin–angiotensin system is placed upstream of SPARC induction, and ADAMTS1 might play roles in mediating the effects of SPARC. In the future, inhibiting the effects of SPARC might be a novel therapeutic strategy for the suppression of hypertensive vascular injury.

## Figures and Tables

**Table 1:** Physiological characteristics of rats treated with deoxycorticosterone acetate and salt for 0 weeks (0w), 1 week (1w), 2 weeks (2w), and 3 weeks (3w).

	0w	1w	2w	3w
Body weight (g)	180 ± 4.6	220 ± 4.9 *	226 ± 8.0 *	218 ± 6.5 *
SBP (mmHg)	119 ± 2.4	139 ± 2.6 *	176 ± 4.1 *#	191 ± 4.6 *#†
HR (bpm)	438 ± 7.3	403 ± 7.2 *	403 ± 5.9 *	420 ± 12
Urinary NOx (μmol/L)	4.79 ± 0.85	4.63 ± 0.55	2.85 ± 0.61	1.70 ± 0.26 *#
Plasma MDA (μmol/L)	17.7 ± 1.2	17.6 ± 1.5	17.6 ± 1.3	21.9 ± 2.1

Values are means ± SEM (*n* = 15–22/group for body weight, SBP, and HR; *n* = 5–6/group for urinary NOx; *n* = 7–10/group for plasma MDA). SBP, systolic blood pressure; HR, heart rate; NOx, NO_2_^−^+NO_3_^−^; MDA, malondialdehyde. * *p* < 0.05 vs. 0w, # *p* < 0.05 vs. 1w, † *p* < 0.05 vs. 2w.

**Table 2:** Primers used for real-time polymerase chain reactions.

Gene Name	Primer Sequences
MCP-1	forward → 5′-TGTTCAGCATTGCTGCCTGT-3′
	reverse → 5′-GATCTCACTTGGTTCTGGTC-3′
LOX-1	forward → 5′-CTCAACTGGAAGCTGAATGG-3′
	reverse → 5′-GGTGGAATGGGAAGTTGCTT-3′
GAPDH	forward → 5′-GGCACAGTCAAGGCTGAGAATG-3′
	reverse → 5′-ATGGTGGTGAAGACGCCAGTA-3′

MCP-1, monocyte chemoattractant protein-1; LOX-1, lectin-like oxidized low-density lipoprotein receptor-1; GAPDH, glyceraldehyde-3-phosphate dehydrogenase.

## Data Availability

The data generated and analyzed during this study are available upon reasonable request from the corresponding author.

## References

[1] Poznyak A.V., Sadykhov N.K., Kartuesov A.G., Borisov E.E., Melnichenko A.A., Grechko A.V., Orekhov A.N. (2022). Hypertension as a risk factor for atherosclerosis: Cardiovascular risk assessment. Front. Cardiovasc. Med..

[2] Rubanyi G.M. (1993). The role of endothelium in cardiovascular homeostasis and diseases. J. Cardiovasc. Pharmacol..

[3] Garcia-Palmieri M.R. (1997). The endothelium in health and in cardiovascular disease. P. R. Health Sci. J..

[4] Herrington W., Lacey B., Sherliker P., Armitage J., Lewington S. (2016). Epidemiology of Atherosclerosis and the Potential to Reduce the Global Burden of Atherothrombotic Disease. Circ. Res..

[5] Brekken R.A., Sage E.H. (2000). SPARC, a matricellular protein: At the crossroads of cell-matrix. Matrix Biol. J. Int. Soc. Matrix Biol..

[6] Sage E.H., Bornstein P. (1991). Extracellular proteins that modulate cell-matrix interactions. SPARC, tenascin, and thrombospondin. J. Biol. Chem..

[7] Toba H., de Castro Bras L.E., Baicu C.F., Zile M.R., Lindsey M.L., Bradshaw A.D. (2015). Secreted protein acidic and rich in cysteine facilitates age-related cardiac inflammation and macrophage M1 polarization. Am. J. Physiol. Cell Physiol..

[8] Ng Y.L., Klopcic B., Lloyd F., Forrest C., Greene W., Lawrance I.C. (2013). Secreted protein acidic and rich in cysteine (SPARC) exacerbates colonic inflammatory symptoms in dextran sodium sulphate-induced murine colitis. PLoS ONE.

[9] Socha M.J., Manhiani M., Said N., Imig J.D., Motamed K. (2007). Secreted protein acidic and rich in cysteine deficiency ameliorates renal inflammation and fibrosis in angiotensin hypertension. Am. J. Pathol..

[10] Rempel S.A., Hawley R.C., Gutierrez J.A., Mouzon E., Bobbitt K.R., Lemke N., Schultz C.R., Schultz L.R., Golembieski W., Koblinski J. (2007). Splenic and immune alterations of the Sparc-null mouse accompany a lack of immune response. Genes. Immun..

[11] Xu L., Ping F., Yin J., Xiao X., Xiang H., Ballantyne C.M., Wu H., Li M. (2013). Elevated plasma SPARC levels are associated with insulin resistance, dyslipidemia, and inflammation in gestational diabetes mellitus. PLoS ONE.

[12] Toba H., Ikemoto M.J., Kobara M., Nakata T. (2022). Secreted protein acidic and rich in cysteine (SPARC) and a disintegrin and metalloproteinase with thrombospondin type 1 motif (ADAMTS1) increments by the renin-angiotensin system induce renal fibrosis in deoxycorticosterone acetate-salt hypertensive rats. Eur. J. Pharmacol..

[13] Matsusaka T., Ichikawa I. (1997). Biological functions of angiotensin and its receptors. Annu. Rev. Physiol..

[14] Dzau V., Braunwald E. (1991). Resolved and unresolved issues in the prevention and treatment of coronary artery disease: A workshop consensus statement. Am. Heart J..

[15] Toba H., de Castro Bras L.E., Baicu C.F., Zile M.R., Lindsey M.L., Bradshaw A.D. (2016). Increased ADAMTS1 mediates SPARC-dependent collagen deposition in the aging myocardium. Am. J. Physiol. Endocrinol. Metab..

[16] Toba H., Jin D., Takai S. (2025). Suppressing SPARC gene with siRNA exerts therapeutic effects and inhibits MMP-2/9 and ADAMTS1 overexpression in a murine model of ischemia/reperfusion-induced acute kidney injury. J. Pharmacol. Sci..

[17] Nakashima Y., Raines E.W., Plump A.S., Breslow J.L., Ross R. (1998). Upregulation of VCAM-1 and ICAM-1 at atherosclerosis-prone sites on the endothelium in the ApoE-deficient mouse. Arterioscler. Thromb. Vasc. Biol..

[18] Sawamura T., Kume N., Aoyama T., Moriwaki H., Hoshikawa H., Aiba Y., Tanaka T., Miwa S., Katsura Y., Kita T. (1997). An endothelial receptor for oxidized low-density lipoprotein. Nature.

[19] Chen M., Masaki T., Sawamura T. (2002). LOX-1, the receptor for oxidized low-density lipoprotein identified from endothelial cells: Implications in endothelial dysfunction and atherosclerosis. Pharmacol. Ther..

[20] Rodriguez-Manzaneque J.C., Milchanowski A.B., Dufour E.K., Leduc R., Iruela-Arispe M.L. (2000). Characterization of METH-1/ADAMTS1 processing reveals two distinct active forms. J. Biol. Chem..

[21] Boring L., Gosling J., Cleary M., Charo I.F. (1998). Decreased lesion formation in CCR2-/- mice reveals a role for chemokines in the initiation of atherosclerosis. Nature.

[22] Yoshimoto R., Fujita Y., Kakino A., Iwamoto S., Takaya T., Sawamura T. (2011). The discovery of LOX-1, its ligands and clinical significance. Cardiovasc. Drugs Ther..

[23] Li X., Zhao W., Li X., Chen X., Li Y., He J., Qin Y., Li L., Zhang H. (2024). The association of SPARC with hypertension and its function in endothelial-dependent relaxation. Atherosclerosis.

[24] Veith C., Varturk-Ozcan I., Wujak M., Hadzic S., Wu C.Y., Knoepp F., Kraut S., Petrovic A., Gredic M., Pak O. (2022). SPARC, a Novel Regulator of Vascular Cell Function in Pulmonary Hypertension. Circulation.

[25] Qi D., Hu X., Wu X., Merk M., Leng L., Bucala R., Young L.H. (2009). Cardiac macrophage migration inhibitory factor inhibits JNK pathway activation and injury during ischemia/reperfusion. J. Clin. Investig..

[26] Workman G., Sage E.H. (2011). Identification of a sequence in the matricellular protein SPARC that interacts with the scavenger receptor stabilin-1. J. Cell. Biochem..

[27] Okada T., Suzuki H., Travis Z.D., Altay O., Tang J., Zhang J.H. (2021). SPARC Aggravates Blood-Brain Barrier Disruption via Integrin alphaVbeta3/MAPKs/MMP-9 Signaling Pathway after Subarachnoid Hemorrhage. Oxid. Med. Cell Longev..

[28] Koepke J.P., Jones S., DiBona G.F. (1986). Renal nerve activity and renal function during environmental stress in DOCA-NaCl rats. Am. J. Physiol..

[29] Shimamura T. (1988). 11-Deoxycorticosterone-induced hypertension, glomerulosclerosis and renal arterial and arteriolar lesions. Jpn. J. Exp. Med..

[30] Michel B., Grima M., Stephan D., Coquard C., Welsch C., Barthelmebs M., Imbs J.L. (1994). Plasma renin activity and changes in tissue angiotensin converting enzyme. J. Hypertens..

[31] Wada T., Kanagawa R., Ishimura Y., Inada Y., Nishikawa K. (1995). Role of angiotensin II in cerebrovascular and renal damage in deoxycorticosterone acetate-salt hypertensive rats. J. Hypertens..

[32] Wu L.L., Cox A., Roe C.J., Dziadek M., Cooper M.E., Gilbert R.E. (1997). Secreted protein acidic and rich in cysteine expression after subtotal nephrectomy and blockade of the renin-angiotensin system. J. Am. Soc. Nephrol..

[33] Toba H., Tojo C., Wang J., Noda K., Kobara M., Nakata T. (2012). Telmisartan inhibits vascular dysfunction and inflammation via activation of peroxisome proliferator-activated receptor-gamma in subtotal nephrectomized rat. Eur. J. Pharmacol..

[34] Toba H., Wang J., Ohigashi M., Kobara M., Nakata T. (2013). Telmisartan protects against vascular dysfunction with peroxisome proliferator-activated receptor-gamma activation in hypertensive 5/6 nephrectomized rats. Pharmacology.

[35] Malaval L., Ffrench M., Delmas P.D. (1990). Circulating levels of osteonectin in normal subjects and patients with thrombocytopenia. Bone Miner..

[36] Malaval L., Fournier B., Delmas P.D. (1987). Radioimmunoassay for osteonectin. Concentrations in bone, nonmineralized tissues, and blood. J. Bone Miner. Res..

[37] Serebruany V.L., Murugesan S.R., Pothula A., Atar D., Lowry D.R., O’Connor C.M., Gurbel P.A. (1999). Increased soluble platelet/endothelial cellular adhesion molecule-1 and osteonectin levels in patients with severe congestive heart failure. Independence of disease etiology, and antecedent aspirin therapy. Eur. J. Heart Fail..

[38] Sandy J.D., Westling J., Kenagy R.D., Iruela-Arispe M.L., Verscharen C., Rodriguez-Mazaneque J.C., Zimmermann D.R., Lemire J.M., Fischer J.W., Wight T.N. (2001). Versican V1 proteolysis in human aorta in vivo occurs at the Glu441-Ala442 bond, a site that is cleaved by recombinant ADAMTS-1 and ADAMTS-4. J. Biol. Chem..

[39] Jonsson-Rylander A.C., Nilsson T., Fritsche-Danielson R., Hammarstrom A., Behrendt M., Andersson J.O., Lindgren K., Andersson A.K., Wallbrandt P., Rosengren B. (2005). Role of ADAMTS-1 in atherosclerosis: Remodeling of carotid artery, immunohistochemistry, and proteolysis of versican. Arterioscler. Thromb. Vasc. Biol..

[40] Okamoto K., Aoki K. (1963). Development of a strain of spontaneously hypertensive rats. Jpn. Circ. J..

[41] Bourd-Boittin K., Bonnier D., Leyme A., Mari B., Tuffery P., Samson M., Ezan F., Baffet G., Theret N. (2011). Protease profiling of liver fibrosis reveals the ADAM metallopeptidase with thrombospondin type 1 motif, 1 as a central activator of transforming growth factor beta. Hepatology.

[42] Francki A., Bradshaw A.D., Bassuk J.A., Howe C.C., Couser W.G., Sage E.H. (1999). SPARC regulates the expression of collagen type I and transforming growth factor-beta1 in mesangial cells. J. Biol. Chem..

[43] Pichler R.H., Hugo C., Shankland S.J., Reed M.J., Bassuk J.A., Andoh T.F., Lombardi D.M., Schwartz S.M., Bennett W.M., Alpers C.E. (1996). SPARC is expressed in renal interstitial fibrosis and in renal vascular injury. Kidney Int..

[44] Toba H., Morishita M., Tojo C., Nakano A., Oshima Y., Kojima Y., Yoshida M., Nakashima K., Wang J., Kobara M. (2011). Recombinant human erythropoietin ameliorated endothelial dysfunction and macrophage infiltration by increasing nitric oxide in hypertensive 5/6 nephrectomized rat aorta. Eur. J. Pharmacol..

